# Green Valorization Strategies of *Pleurotus ostreatus* and Its By-Products: A Critical Review of Emerging Technologies and Sustainable Applications

**DOI:** 10.3390/molecules30214318

**Published:** 2025-11-06

**Authors:** Pablo Ayuso, Jhazmin Quizhpe, Rocío Peñalver, Pascual García-Pérez, Gema Nieto

**Affiliations:** Department of Food Technology, Nutrition and Food Science, Veterinary Faculty, Campus de Excelencia Internacional de Ámbito Regional (CEIR) Campus Mare Nostrum (CMN), University of Murcia, 30100 Murcia, Spain; pablo.ayuson@um.es (P.A.); jhazminedith.quizhper@um.es (J.Q.); rocio.penalver@um.es (R.P.); pascual.garcia@um.es (P.G.-P.)

**Keywords:** oyster mushroom, β-glucans, functional foods, by-products valorization, green extraction technologies, dietary fiber

## Abstract

*Pleurotus ostreatus*, commonly known as the oyster mushroom, is a widely cultivated edible mushroom characterized by its nutritional value and health benefits. However, its large-scale production generates significant amounts of agro-industrial by-products, such as stipes, residual mycelium, and spent mushroom substrate (SMS). These by-products are often discarded despite their high content of bioactive compounds such as dietary fiber, β-glucans, polyphenols, ergosterol, and essential minerals. This review provides a critical overview of emerging green extraction technologies (i.e., ultrasound-assisted extraction (UAE), microwave-assisted extraction (MAE), supercritical fluid extraction (SFE), subcritical water extraction (SWE), enzyme-assisted extraction (EAE), and pulsed electric fields (PEF)) as a strategy for the sustainable valorization of bioactive compounds from *P. ostreatus* by-products. Despite promising results in the extraction of β-glucans and phenolic compounds, industrial scalability remains a challenge due to cost, energy demand, and regulatory issues. In addition, the potential incorporation of these compound by-products into functional food formulations is explored, highlighting their possible applications in meat, bakery, and dairy products. Although notable outcomes have been obtained in the use of the fruiting body as a functional ingredient, further research is needed into the potential use of by-products in order to optimize processing parameters, ensure safety, and validate consumer acceptance. Overall, the sustainable valorization of *P. ostreatus* by-products represents a dual opportunity to reduce food waste and develop innovative functional ingredients that contribute to health and sustainability.

## 1. Introduction

**Figure 1:**
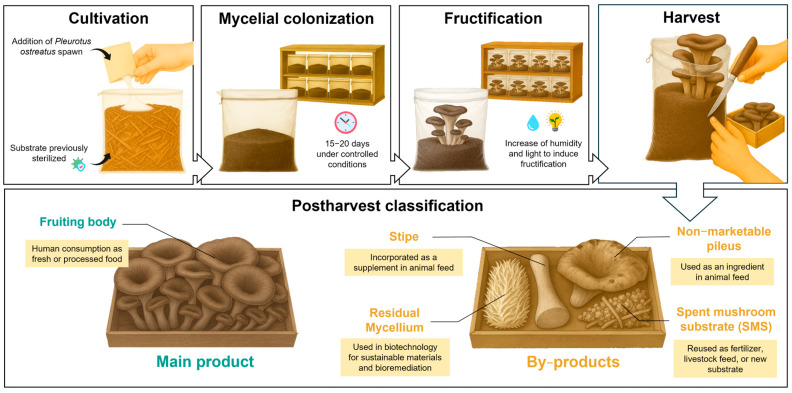
Cultivation process and postharvest classification of *Pleurotus ostreatus* and its by-products.

*Pleurotus ostreatus* (Jacq.) P. Kumm, commonly known as the oyster mushroom, is a species belonging to the Basidiomycota phylum. This mushroom is considered highly attractive due to its low production cost and strong adaptability to various environmental conditions [1]. *P. ostreatus* is easily cultivated on lignocellulosic substrates, previously pasteurized or sterilized, under controlled temperature and humidity conditions that favor the growth of mycelium and the formation of fruiting bodies (Figure 1). The edible part, or fruiting body, is considered a healthy food because it is rich in a variety of bioactive compounds, including β-glucans, polyphenols, and essential minerals [2]. Numerous studies have highlighted the antioxidant [3], immunomodulatory [4], and hypocholesterolemic [5] activity of oyster mushrooms.

The edible mushroom market has experienced rapid expansion, reaching a market value of USD 62.19 billion in 2023, with an estimated global production of 20.84 million tons in 2026 [6]. Of this total production, the genus *Pleurotus* spp. accounts for approximately 27% of all edible mushrooms produced [7], only surpassed by *Agaricus bisporus*. As a result, edible mushroom production generates large amounts of by-products, estimated at 5 kg of by-products for every kg of fresh mushrooms [8]. The agrifood by-products of *P. ostreatus* can be divided into stipe, non-marketable pileus, residual mycelium, and spent mushroom substrate (SMS) (Figure 1). These residual biowastes have been reported to provide significant amounts of bioactive compounds and essential nutrients, with particular emphasis on their role as sources of dietary fiber, glucans, and polyphenols.

Currently, there is growing interest in the valorization of these by-products, as they have no clear commercial value. The compounds of interest in oyster mushrooms have usually been extracted using conventional techniques such as maceration extraction, reflux extraction (Soxhlet), or infusion extraction. However, these methods have a long extraction time, limited efficiency, and require the use of a large amount of solvents. For these reasons, numerous innovative and environmentally friendly extraction technologies have emerged as an effective alternative to conventional methods [9]. However, the use of green technologies such as ultrasound-assisted extraction (UAE), microwave-assisted extraction (MAE), supercritical fluid extraction (SFE), or subcritical water extraction (SWE) represents a challenge for industrial application due to their current cost and performance [10], which is why further research is needed on their potential use in the recovery of plant or fungal matrices. On the other hand, the possibility of valorizing food by-products as ingredients for functional foods is being explored, representing a promising strategy that not only adds nutritional and health-promoting properties to the human diet but also contributes to the sustainability of food systems [11]. These strategies could reduce food waste, improve resource efficiency, and align with the United Nations Sustainable Development Goals (SDGs).

In this review, different emerging green technologies will be examined as a pathway for valorizing oyster mushrooms and their agro-industrial by-products. In addition, special emphasis will be placed on integrating these extraction methods with the development of functional food ingredients, highlighting how this combined strategy can maximize nutritional and health benefits while promoting sustainable food systems. This review aims to provide a critical overview of the dual approach of green extraction and functional valorization, underlining its relevance for both scientific research and industrial application.

## 2. Pleurotus Ostreatus By-Products: Composition and Potential

### 2.1. Fruiting Body

The fruiting body is the edible and globally commercialized part of *P. ostreatus*. It is generally composed of the pileus (cap) and the stipe (stem). In recent years, it has gained increasing attention due to its remarkable nutritional and functional profile, emphasizing its potential as a source of bioactive compounds with antioxidant, anti-inflammatory, hypolipidemic, and immunomodulatory properties [12]. The fruiting body of *P. ostreatus* represents a good source of total dietary fiber (TDF) (Table 1), consisting of approximately 84% insoluble dietary fiber (IDF) and 16% soluble dietary fiber (SDF) [13]. Notably, SDF exerts notable health benefits, as it reduces glycemic response, lowers blood cholesterol and blood pressure, prevents gastrointestinal problems, and provides protection against certain types of cancer [14]. Oyster mushroom also contains a notable amount of protein (17–23.9 g/100 g dry weight (DW)), with the branched chain amino acids (BCAA) (leucine, valine, and isoleucine) being the most abundant. Majesty et al. [15] reported that the essential amino acids of *P. ostreatus* reached values of 42.51%, suggesting a substantial supply for humans. Additionally, oyster mushroom presents a very low crude fat content (2.5–2.6 g/100 g DW), being especially rich in monounsaturated fatty acids (MUFA) such as oleic acid and polyunsaturated fatty acids (PUFA) such as linoleic acid [12]. It is important to mention that the fruiting body and cap of *P. ostreatus* are distinguished by their high moisture content (88.8–93 g/100 g FW), which dilutes their content in the macronutrients previously described.

A large part of the fruiting body of *P. ostreatus* is composed of carbohydrates (37.8 g/100 g DW), with glucose (55.1 g/100 g DW) being the most abundant monosaccharide, followed by galactose (17.4 g/100 g DW). However, its β-glucan content (29.9 g/100 g DW) stands out as the most relevant bioactive fraction. β-glucans are a heterogeneous group of glucose polymers with a typical structure comprising a main chain of β-(1,4) and/or β-(1,3)-glucopyranosyl units with various branching and lengths as side chains. Among them, pleuran (β-(1,3/1,6)-D-glucan) is the specific β-glucan of *P. ostreatus* and the most studied in this species. These non-starch polysaccharides, present in fungi, yeasts, and some algae, have been widely associated with certain health benefits, including immunomodulatory and antioxidant effects. In addition, the prebiotic potential of β-glucans has been recognized, as they may benefit the gut microbiota by acting as prebiotic substrates that selectively stimulate the growth of beneficial bacteria. The fruiting body of oyster mushrooms stands out as a rich source of essential minerals, with a remarkable content of macrominerals such as potassium, phosphorus, magnesium, sodium, and calcium (Table 1). Specifically, potassium and phosphorus are the two most abundant macrominerals, which play a key role in nerve control and bone formation, respectively [16,17]. Among the trace minerals, iron, copper, and manganese are also found in relevant quantities. These trace minerals are vital for immune function, antioxidant protection, and protein synthesis. In addition, Fazlić et al. [18] studied the mineral profile of several mushroom species, reporting similar results between oyster mushroom and pink oyster, lion’s mane, or reishi species. *P. ostreatus* is also a valuable source of vitamins, which contribute to its nutritional and functional potential. Among the water-soluble vitamins, riboflavin (B2) is the most abundant (0.58 g/100 g DW), followed by thiamine (B1, 0.32 g/100 g DW) [19]. In terms of fat-soluble vitamins, α-tocopherol (25.54 mg/100 g DW) stands out [20], which plays a key role in cardiovascular and immune health due to its high antioxidant activity [21].

Phenolic compounds represent another important class of bioactive constituents in the oyster mushroom, widely recognized for their antioxidant, anti-inflammatory, and antimicrobial activities. Phenolic acids are the most important polyphenol family in this species, with homogentisic acid (629.86 μg/g DW) and gentisic acid (292.62 μg/g DW) being the most abundant [22]. In addition, Koutrotsios et al. [23] reported the presence of other phenolic acids such as syringic acid, vanillic acid, or caffeic acid in *P. ostreatus*. Numerous flavonoids have also been described in oyster mushrooms, with chrysin (40.0 mg/100 g DW) and rutin (31.2 mg/100 g DW) being the most representative [24]. However, the concentration of these compounds can vary considerably depending on substrate composition, temperature, or harvesting.

### 2.2. Stipe

The stipe of *P. ostreatus* is a structural component of the fruiting body that provides mechanical support to the pileus and facilitates spore dispersal. Both the stipe and pileus develop from the mycelial network, which is the actual structure responsible for nutrient absorption and transformation from the substrate. It is considered part of the fruiting body, representing about 30% of its total mass. However, this structure is often removed during food processing because it is difficult to chew and swallow and has a low absorption rate. In addition, stipes can constitute up to 20% of the total production yield [25], which is why new approaches for their valorization are increasingly being explored. *P. ostreatus* stipe shows a high content of health-promoting bioactive compounds. Golian et al. [26] studied the fiber content of 60 different strains of *P. ostreatus*, reporting that the content in the stipe was higher than pileus in terms of IDF, SDF, and TDF, reaching values up to 64.8 g/100 g DW of total fiber. On the other hand, this research also demonstrated a high content of β-glucans from this by-product, being up to 33% higher than those found in the cap. Pérez-Bassart et al. [27] found antiviral activity of β-glucans from oyster mushroom stipes against murine norovirus, due to the higher ramification of the β-glucans present in this by-product. It should be noted that the stipe presents a similar amount of α-tocopherol and α-tocotrienol as the fruiting body of *P. ostreatus*, and these compounds are very important in the nutritional characteristics of this species. Moreover, Diamantopoulou et al. [2] observed a notable amount of δ-Tocopherol in the stipe of oyster mushroom (0.42 mg/kg DW), while it was not detected in the fruiting body or cap. This isoform of tocopherol has been associated with relevant bioactivities, including the inhibition of apoptosis and autophagy in colon cancer models and in HepG2 hepatocarcinoma cells [28]. Finally, a high content of key vitamins such as riboflavin (Vit. B2) has been found in stipe, with stipe containing up to 3 times more riboflavin than *P. ostreatus* cap [29].

However, the oyster mushroom stipe contains a lower content of minerals compared to the pileus. Cherno et al. [30] reported a lower content of macroelements (K, Ca, Na, Mg, P) as well as trace elements (Zn, Se, Fe, Mn) in the stipe. The highest reduction was seen in phosphorus, where the fruiting body reached up to twice the content. In addition, stipe exhibits a lower protein content than pileus, especially essential amino acids such as isoleucine, lysine, threonine, tryptophan, or valine (Table 1).

Despite the considerable nutritional power of *P. ostreatus* stipes, they are generally discarded during processing, and their potential revalorization remains unexploited. Although the stipe is the most studied by-product of oyster mushrooms, no data are currently available regarding the individual phenolic compounds present in this botanical part, which could have key roles in its valorization. Nevertheless, it is known that its antioxidant capacity and total phenolic content are lower than those found in pileus (Table 1).

### 2.3. Spent Mushroom Substrate (SMS)

Spent mushroom substrate (SMS) is the soil-like organic residue or by-product remaining after mushroom harvesting. It is the material that remains after the mushroom production cycle and is no longer suitable for mushroom cultivation. SMS consists of a mixture of spent lignocellulosic material (i.e., wheat, sawdust, rice straw, and corn cobs), organic materials (i.e., proteins, carbohydrates), mycelium, and nutrients not consumed by the mushroom. It is the main by-product of *P. ostreatus*, estimating that for each kilogram of mushrooms produced, approximately 5 kg of SMS is generated as a by-product [31].

Despite being considered a biowaste from mushroom cultivation, the SMS of *P. ostreatus* contains essential nutrients. In particular, high dietary fiber content has been reported in SMS (30.44 g/100 g DW), similar to that found in the fruiting body. This amount of TDF can be attributed to the fibrous content of the substrates used during cultivation. In addition, high levels of lignocellulosic components such as cellulose (35.95 g/100 g DW), hemicellulose (13.14 g/100 g DW), and lignin (6.59 g/100 g DW) have been reported. These insoluble fibers serve as a source of carbon for reuse in mushrooms, but they can also affect intestinal functions and modulate nutrient digestion and passage rate at a moderate level in the diet [32]. In addition, *P. ostreatus* SMS is a good source of minerals. Zakil et al. [33] reported a significant concentration of macroelements such as Ca and Mg, as well as essential trace elements such as Fe and Mn, which were even higher than those found in the fruiting body (Table 1). SMS retains a significant proportion of the minerals originally present in the culture substrate (straw, sawdust, agricultural waste, etc.), since oyster mushroom only absorbs the fraction it needs for its metabolism and formation. On the other hand, recent studies have investigated the bioactive and antioxidant potential of oyster mushroom SMS. Significant amounts of chlorogenic acid, caffeic acid, p-coumaric acid, ferulic acid, gallic acid, and vanillic acid have been detected in SMS [34]. However, the concentrations of these phenolics are highly dependent on the extraction method used.

In recent years, several options for SMS valorization have emerged through various applications. Efforts have been made to use SMS as a cost-effective source of ligninolytic enzymes for industrial purposes. Fungal enzymes, such as laccase and peroxidases, have been successfully extracted for this biotechnological purpose. In addition, several studies have demonstrated the viability of using SMS in horticultural applications, as its application alters soil structure and porosity, increases mineral nitrogen content in the soil, and can act as a biological control [35]. However, SMS is not currently revalued and is usually disposed of by spreading it on the ground, depositing it in landfills, or composting it with livestock manure.

**Table 1:** Proximate composition, bioactive compounds, and micronutrients of main products and agro-industrial by-products of *P. ostreatus*.

Composition		Main Products		By-Products			Refs.
		Fruiting Body	Pileus	Stipe	SMS	Mycelium	
Moisture	(g/100 g FW)	88.8	93	92.2	15.6	18	[2,36,37]
Ash	(g/100 g DW)	4.9	5.2	3.4	5.3	12	[2,36,37]
Protein	(g/100 g DW)	23.9	17	10	7.5	5	[36,38,39,40]
Fat	(g/100 g DW)	2.6	2.5	2	7.5	0.1	[30,36,40,41]
TDF	(g/100 g DW)	32.7	34.5–63.1	38.9–64.8	30.4	55	[13,36,42,43]
IDF	(g/100 g DW)	27.58	29.2–51.4	36.7–61.4	-	22	[13,36,42,43]
SDF	(g/100 g DW)	5.14	2.0–4.7	3.3–4.9	-	33	[13,36,42,43]
Carbohydrates	(g/100 g DW)	37.8	3.5	4.4	30.5	61	[36,37]
Galactose	(g/100 g DW)	17.4	1	0.5	-	-	[1,30]
Glucose	(g/100 g DW)	55.1	82.5	88.5	-	-	[1,30]
Xylose	(g/100 g DW)	7.2	5	5	-	-	[1,30]
α-glucans	(g/100 g DW)	1	1.4	2.2	-	-	[26,44,45,46]
β-glucans	(g/100 g DW)	29.9	36	48	-	39.5	[26,44,45,46]
Total glucans	(g/100 g DW)	29.2	38	50	-	-	[26,44,45,46]
Free Amino Acids							
Alanine	(mg/g DW)	7.22	1.16	0.43	-	-	[30,47]
Aspartic acid	(mg/g DW)	2.51	1.94	1.04	-	-	[30,47]
Glutamic acid	(mg/g DW)	4.81	3.26	1.72	-	-	[30,47]
Glycine	(mg/g DW)	1.03	0.73	0.4	-	-	[30,47]
Histidine	(mg/g DW)	0.88	0.36	0.21	-	-	[30,47]
Isoleucine	(mg/g DW)	2.8	0.63	0.38	-	-	[30,47]
Leucine	(mg/g DW)	8.59	1.14	0.92	-	-	[30,47]
Lysine	(mg/g DW)	2.47	0.95	0.6	-	-	[30,47]
Methionine	(mg/g DW)	1.14	0.39	0.19	-	-	[30,47]
Phenylalanine	(mg/g DW)	2.95	0.73	0.5	-	-	[30,47]
Proline	(mg/g DW)	1.21	0.70	0.57	-	-	[30,47]
Serine	(mg/g DW)	3.93	0.92	0.52	-	-	[30,47]
Threonine	(mg/g DW)	2.29	0.77	0.37	-	-	[30,47]
Tryptophan	(mg/g DW)	1.07	0.22	0.14	-	-	[30,47]
Tyrosine	(mg/g DW)	2.66	0.60	0.49	-	-	[30,47]
Valine	(mg/g DW)	4.24	0.92	0.59	-	-	[30,47]
Macroelements							
K	(mg/Kg DW)	37,300	30,741	21,389	9000	-	[33,48]
Ca	(mg/Kg DW)	260	820	853	22,800	-	[33,48,49]
Na	(mg/Kg DW)	615	269	265	100	-	[33,48,49]
Mg	(mg/Kg DW)	2000	1369	933	4100	-	[33,48]
P	(mg/Kg DW)	13,900	6983	2950	2300	-	[33,48]
Trace elements							
Zn	(mg/Kg DW)	38	76.6	36.5	20	-	[19,33,48]
Se	(mg/Kg DW)	0.1	nd	nd	-	-	[19,48]
Fe	(mg/Kg DW)	15.8	156.0	120.0	200	-	[19,33,48]
Mn	(mg/Kg DW)	11.0	9.6	6.2	150	-	[33,48]
Cu	(mg/Kg DW)	13.5	18.7	20.2	-	-	[48,50]
Mo	(mg/Kg DW)	0.0	0.0	0.4	-	-	[48,51]
Cr	(mg/Kg DW)	0.1	0.9	0.8	-	-	[48,51]
Vitamins							
α-Tocopherol	(mg/Kg DW)	0.78	0.79	0.67	-	-	[2]
α-Tocotrienol	(mg/Kg DW)	0.28	0.26	0.30	-	-	[2]
β-Tocotrienol	(mg/Kg DW)	0.27	0.27	0.15	-	-	[2]
δ-Tocopherol	(mg/Kg DW)	nd	nd	0.42	-	-	[2]
Phenolic content and antioxidant activity							
TPC	(mg GAE/g)	24	25.9	11.3	9.1	-	[2,34]
DPPH	(mg TE/g)	2.48	2.09	0.44	-	-	[2]
ABTS	(mg TE/g)	3.21	2.97	2.17	-	-	[2]
FRAP	(mg TE/g)	3.26	2.92	0.25	-	-	[2]

Values correspond to individual data reported in the cited references. When multiple values were available for the same parameter, one representative value was selected based on sample comparability and methodological consistency. Abbreviations: ABTS: 2,2′-azino-bis(3-ethylbenzothiazoline-6-sulfonic acid); Ca: calcium; Cr: chromium; Cu: copper; DPPH: 2,2-diphenyl-1-picrylhydrazyl; DW: dry weight; Fe: iron; FRAP: ferric reducing antioxidant power; FW: fresh weight; GAE: gallic acid equivalents; IDF: insoluble dietary fiber; K: potassium; Mg: magnesium; Mn: manganese; Mo: molybdenum; Na: sodium; nd: not detected; P: phosphorus; Se: selenium; SDF: soluble dietary fiber; TDF: total dietary fiber; TE: Trolox equivalents; TPC: total phenolic content; Zn: zinc.

### 2.4. Residual Mycelium

Residual mycelium is the fungal biomass that remains immersed in the soil once the main production cycle and fruiting body harvest are complete. It is mainly composed of vegetative mycelial tissue that has not developed and is often mixed with spent mushroom substrate residues. Although the production volume is lower than that of SMS or stipe, it is a secondary by-product with potential applications due to its nutritional and functional characteristics.

Specifically, this by-product is a great source of dietary fiber. Laforteza et al. [36] reported values of 55 g/100 g DW of TDF in this anatomical part of the oyster mushroom, being higher than that reported by other studies in the SMS or fruiting body. It should also be noted that these researchers observed much higher SDF results than any other by-product of this species, reaching a content of 33 g/100 g DW. Moreover, the mycelium of *P. ostreatus* is also a good source of carbohydrates, especially β-glucans. Values of up to 39.5 g/100 g of β-glucans have been reported in this by-product, which is higher than that found in the fruiting body and slightly lower than that found in the stipe (Table 1). The mycelium of *P. ostreatus* has also been described as a great source of ergothioneine [52], an amino acid derived from histidine with a powerful antioxidant and cytoprotective effect, capable of neutralizing reactive oxygen species (ROS) and regulating Nrf2 activity. In the diet, ergothioneine contributes to protection against oxidative stress, inflammation, and cellular damage. In addition, recent research has associated ergothioneine with reduced cognitive decline, reduced cardiovascular risk, and improved aging [53]. This compound is found in greater quantities in the mycelium than in the fruiting body of the oyster mushroom, as the residual mycelium is exposed to more metabolic and oxidative stress, causing it to accumulate ergothioneine as a defense mechanism.

Currently, residual mycelium from oyster mushroom cultivation has no valuable applications and is considered a waste by-product. However, some research has attempted to use this mycelium as a biomaterial for the textile industry due to its highly fibrous and intertwined structure [54,55]. Nevertheless, this agro-industrial by-product has been poorly studied, and no study has reported information about bioactive compounds such as vitamins or phenolic compounds. Furthermore, from a physiological perspective, residual mycelium is vegetative tissue of the mushroom, similar to the mycelium that forms the fruiting body, and could therefore be the by-product most similar nutritionally to the commercial oyster mushroom product. This underscores the need for more in-depth research into this agricultural waste.

## 3. Application of Green Technologies for *P. ostreatus* Valorization

The application of green extraction technologies represents a crucial step in the valorization of *Pleurotus ostreatus* and its by-products. These methods not only enable the recovery of bioactive compounds, such as phenolic acids, β-glucans, ergosterol, polysaccharides, peptides, and tocopherols, but also determine their functional potential and suitability for incorporation into food matrices. The extraction process directly influences the physicochemical stability, solubility, and bioavailability of these molecules, which are essential parameters for developing functional ingredients and fortifying foods with enhanced nutritional and technological properties. In this sense, the choice of an appropriate extraction strategy is not only a technological decision but also a determinant of the functional efficacy of the recovered compounds in real food systems. The following subsections summarize the main green extraction technologies currently explored for *P. ostreatus* valorization, emphasizing their compound specificity, extraction yields, and their potential translation to food applications.

### 3.1. Ultrasound-Assisted Extraction (UAE)

Ultrasound-assisted extraction (UAE) is a green extraction technique that applies ultrasonic energy and solvents to extract specific compounds from plant or fungal matrices. This technique uses ultrasonic waves (>20 kHz) to cause cavitation bubbles that collapse, producing mechanical effects such as fragmentation, localized erosion, pore formation, shear forces, and increased permeability of cell walls, thereby facilitating the release of bioactive compounds into the solvent (i.e., acidified water, ethanol, alcohols, acetone, water) [56]. UAE has been successfully used to recover a wide range of bioactive compounds from food by-products. This technique has proven effective to extract phenolic compounds from citrus peels, apple pomace, and flax seeds [57], as well as natural pigments such as carotenoids, anthocyanins, and betalains from fruit and vegetable residues [58]. In addition, the UAE has also been effective in recovering pectins from grape pomace, jackfruit peel, and pomegranate peel [59].

UAE has been applied for the extraction of various bioactive compounds in *P. ostreatus* by-products, particularly in the valorization of SMS (Table 2). Estefes-Duarte et al. [60] studied the application of different wave amplitudes, times, and solvent concentrations with the aim of optimizing the extraction of antioxidant compounds in SMS using this technique. This study concluded that the most important parameter for SMS extraction was wave amplitude, where a wave amplitude of 100% proved optimal for the extraction of phenols, flavonoids, and ABTS antioxidant activity. However, tannins were most effectively extracted with a wave amplitude of 20%. Another study investigated the ability to extract phenolic compounds from SMS using UAE as well as reflux or Soxhlet extraction [34]. SMS extraction using UAE (temperature: 65 °C, time: 60 min) obtained the best extraction yield (11.1%) and was able to efficiently extract carbohydrates (7.3 g/L). However, conventional Soxhlet extraction achieved better results than UAE in the extraction of total phenolic compounds. Nevertheless, this technique allowed for the efficient extraction of phenolic compounds such as chlorogenic, p-coumaric, or ferulic acids. Ultrasound-assisted extraction has also been used in other by-products of *P. ostreatus*. Prandi et al. [61] studied how different extraction methods behaved in the extraction of peptides from processing cuttings, leftovers, and mycelium. UAE offered good extraction efficiency, but the heat generated during sonication caused racemization of sensitive compounds, so it is necessary to control the temperature to prevent degradation.

**Figure 2:**
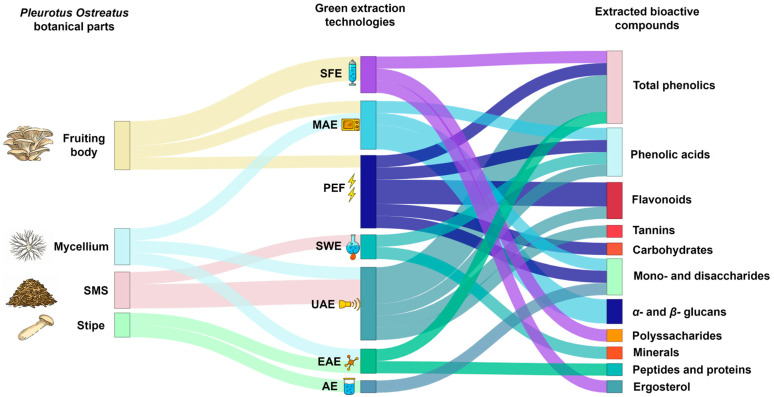
Sankey diagram of green extraction technologies applied to *P. ostreatus* by-products and their associated bioactive compounds. Source: [34,44,60,61,69,70,71,72,73,74,75,76,77]. The thickness of each connection represents the number of studies supporting each association between extraction technique and recovered compounds.

Overall, the UAE has proven to be an interesting alternative to conventional extraction methods and a way to valorize *P. ostreatus* by-products. However, this methodology presents certain limitations, as the use of high temperatures during extraction can degrade thermolabile compounds such as phenolic acids and vitamins. In addition, studies showed low extraction yields (17% and 11.1%) and a need to optimize parameters depending on the substrate and compound to be extracted. In addition, its potential is currently limited to the recovery of antioxidant compounds (Figure 2). Nevertheless, studies conducted on other mushroom species, such as shiitake [62,63], have demonstrated the potential of UAE to extract heteropolysaccharides (including L-rhamnose, D-glucuronic acid, D-galacturonic acid, D-glucose, and D-xylose), as well as mycosterols such as ergosterol, ergosta-7,22-dienol, and β-sitosterol, which are key in mushroom valorization. Finally, only the use of UAE in SMS and mycelium has been explored. Future research should address its potential for soluble fiber extraction, particularly for the recovery of β-glucans in the stipe of oyster mushrooms. This extraction approach has been applied to other species, such as *Lentinula edode* or *Agaricus bisporus* [64,65].

### 3.2. Microwave-Assisted Extraction (MAE)

Microwave-assisted extraction (MAE) is an environmentally friendly and efficient extraction technique that uses a combination of microwave energy and polar solvents (i.e., water, ethanol), causing rapid heating and pressure within plant cells. This leads to cell wall rupture and increased solvent penetration, thereby releasing the target compounds into the solvent. This innovative extraction method has been used to recover intracellular compounds of interest in food by-products, including antioxidants and polyphenols [66], essential oils [67], and oligo/polysaccharides [68].

This extraction technique is also promising for extracting bioactive compounds from oyster mushrooms. In a recent study, MAE was validated for profiling phenolic compounds in a selection of oyster mushrooms from various geographical regions [69]. Box–Behnken design and response surface methodology reported that a microwave power of 800 W, a 17.5:1 mL/g solvent-to-sample ratio, and a temperature of 44 °C for 10 min were able to efficiently extract cinnamic acid, p-coumaric acid, p-hydroxybenzoic acid, and gallic acid from American, Spanish, and Portuguese *P. ostreatus* varieties. Notably, phenol recovery using this methodology yielded very high values (>85%), especially for cinnamic acid (97.07%) and p-hydroxybenzoic acid (97.74%), indicating that MAE is a great alternative for phenol recovery in *P. ostreatus*. However, due to the ability of MAE to disrupt the cell wall due to the rapid heating of microwaves, this technique is particularly valuable for the extraction of soluble fibers and polysaccharides from mushrooms. Specifically, this technique has been used to recover monosaccharides such as glucose, mannose, and galactose, as well as β-d-glucans in the fruiting body of *P. ostreatus* [70]. Furthermore, this study obtained good yields (32.4% of total yield and 16.5% of carbohydrates) using standard extraction conditions (850 W, 2455 MHz). Nevertheless, few studies have used MAE for the extraction of bioactive compounds in oyster mushroom by-products. This technology could be particularly relevant for the revalorization of biowaste such as stipe or mycelium, due to their high soluble fiber and beta-glucan content. Only one study evaluated the extraction capacity of MAE in oyster mushroom mycelium, obtaining good results in the extraction of polysaccharides [44]. This study showed that MAE was more effective in the extraction of β-glucans than other conventional techniques based on extraction with water and ethyl alcohol, increasing the content of these soluble polysaccharides by up to 60%.

**Table 2:** Recovery of bioactive compounds from *P. ostreatus* using green extraction technologies.

Botanical Part	Extraction Technique	Conditions	Yield (%)	Extracted Compounds/Outcomes	Refs.
Fruiting Body	PEF	200 mL water, specific energy 50 kJ/kg, 2.5 kV/cm, 6 h stirring	Not reported	Cinnamic acid, hesperidin, quercetin, carbohydrates, minerals (Mg, Fe, P, K)	[71,72]
				Increased antioxidant activity (ORAC, TEAC, TPC)	
Fruiting Body	MAE	800 W, 10 min, temperature range 40–70 °C	86.3–97.7	Cinnamic acid, p-coumaric acid, p-hydroxybenzoic acid, gallic acid	[69]
Fruiting Body	SFE	15–25 MPa, 40–60 °C, 90 min (10 min static + 80 min dynamic), CO_2_ flow 20 L/h, ethanol co-solvent (100–200 mL)	Not reported	Ergothioneine, polyphenols	[73]
Fruiting Body	SFE	120–160 °C, 15–35 MPa, 10–20% H_2_O, CO_2_ flow 2.5 L/min, 120 min dynamic extraction, sample/glass bead ratio 1:2	30.69	Heteropolysaccharides, β-glucans, α-glucans, oligosaccharides	[74]
Fruiting Body	SFE	40 °C, 30 MPa, CO_2_ flow 1.94 kg/h, 30–240 min dynamic extraction, separator 1.5 MPa/23 °C	0.8	Fatty acids, sterols	[75]
Fruiting Body	MAE	850 W, 2455 MHz	32.4	Glucose, mannose, galactose, β-glucans	[70]
Stipe	EAE	Protamex^®^ 2%, pH 7, 55 °C for 3 h + V8 protease 0.06%, pH 7.8, 37 °C for 24 h	84	Peptides < 1 kDa, peptides 1–3 kDa	[76]
Stipe	AE	3 g mushroom powder heated in 250 mL water at 80 °C for 3 h	35	Galactose, mannose	[77]
SMS	UAE	Amplitude 20–100%, time 5–15 min, 10–30% methanol concentration	Not reported	Total polyphenols, flavonoids, tannins	[60]
				Enhanced antioxidant activity (DPPH, ABTS)	
SMS	UAE	Temperature 35–65 °C, time 30–60 min, solvent 40:60 (*v*/*v*) ethanol/water, 20 kHz, 250 W power, 50% amplitude	11.1	Total polyphenols, total carbohydrates	[34]
SMS	SWE	150 °C for 10 min	16.3	Total carbohydrates, caffeic acid, gallic acid, vanillic acid	[34]
Mycelium	MAE	800 W, 2450 MHz frequency	21	β-glucans	[44]
Cuttings, Leftovers, Mycelium	UAE	400 W, 20 kHz, 30 g dried mushroom by-product, 0.1 M NaOH, temp < 45 °C,	17	Protein extracts	[61]
Cuttings, Leftovers, Mycelium	EAE	Protease from *Bacillus licheniformis* (pH 6.5–8.5, 60 °C) or papain (pH 6.0–7.0, 65 °C), 1% enzyme-to-substrate ratio, 2 h hydrolysis	23–24	Protein extracts	[61]

Abbreviations: ABTS: 2,2′-azino-bis(3-ethylbenzothiazoline-6-sulfonic acid) assay; AE: aqueous extraction; EAE: enzyme-assisted extraction; Fe: iron; K: potassium; MAE: microwave-assisted extraction; Mg: magnesium; ORAC: oxygen radical absorbance capacity; P: phosphorus; PEF: pulsed electric fields; SFE: supercritical fluid extraction; SWE: subcritical water extraction; TPC: total phenolic content; TEAC: Trolox equivalent antioxidant capacity; UAE: ultrasound-assisted extraction.

### 3.3. Supercritical Fluid Extraction (SFE)

Supercritical fluid extraction (SFE) is an innovative technology that is increasingly used in the extraction of by-products from plant or food matrices. This technique uses a fluid, usually CO_2_, above its critical temperature and pressure, which has a diffusivity similar to that of a gas and a solvation power similar to that of a liquid. As a result of its properties, this supercritical fluid can easily penetrate the food matrix and allow for the efficient isolation of high-quality products with minimal damage and residual content that dissolves the target bioactive compounds and transports them to a separator where a reduction in pressure precipitates the extracted compounds. This technique has been widely used in various plant matrices, mainly to extract lipophilic compounds (i.e., essential oils, carotenoids, and vitamins) and moderately polar compounds, such as phenolic compounds, chlorophylls, or xanthophylls, if ethanol is used as a co-solvent [78].

This technique has been of particular interest for the extraction of different bioactive compounds from oyster mushrooms. Specifically, SC-CO_2_-SFE combined with response surface methodology has been effective in the recovery of ergothioneine and polyphenols [73]. In this study, the optimized SFE technique (21 MPa, temperature of 48 °C, and co-solvent amount of 133 mL) was able to extract 1.35 mg/g DW of ergothioneine and a total phenol content of 5.48 mg GAE/g DW. Another study applied SFE and water as a co-solvent (H_2_O + CO_2_-SFE) to obtain extracts rich in antioxidant polysaccharides from the fruiting body of *P. ostreatus* [74]. This combination demonstrated good yields in the extraction of heteropolysaccharides, as well as β-glucans and α-glucans. Nevertheless, the main use of SFE in food matrices is in the extraction of lipids. A recent study investigated how SFE affected the extraction of fatty acids and sterols in five different mushroom species, including *P. ostreatus* [75]. In this study, oyster mushrooms had the lowest total yield values after extraction compared to other species, mainly because the fruiting body of this species has a very low lipid content (2.6 g/100 g DW, Table 1). However, this study demonstrated that SFE is a very effective technique for the extraction of ergosterol, with *P. ostreatus* showing the highest recovery of this compound. Ergosterol is a key compound in *P. ostreatus*, as it is the main sterol present in mushrooms and has been associated with antioxidant, anti-inflammatory, and immunomodulatory properties, as well as playing an important role as a D_2_ vitamin precursor [79].

However, the application of SFE in mushrooms has some limitations, especially for the extraction of polar compounds, such as phenols and polysaccharides, where techniques such as MAE and UAE may be more effective. Nevertheless, SFE is very powerful for recovering lipophilic bioactives, such as fatty acids, sterols, and fat-soluble vitamins, with minimal thermal degradation. It is important to mention that no study has applied SFE to valorize the by-products of *Pleurotus ostreatus*, which represents an innovative and unexploited opportunity. For example, although ergosterol is more abundant in the fruiting body, SFE could efficiently extract it from non-marketable pileus, converting residues into a source of bioactive sterols. Similarly, fat-soluble vitamins, such as α-, β-, and γ-tocopherols, could be recovered from the stipe, whose content is similar to that of the fruiting body (Table 1).

### 3.4. Enzyme-Assisted Extraction (EAE)

Enzyme-assisted extraction (EAE) is a technique that involves the use of different specific enzymes to decompose the structural components of the plant or fungi matrix, such as cell walls, pectins, cellulose, or hemicellulose, thus facilitating the extraction of intracellular compounds of interest. It is considered an environmentally friendly technique since it does not require the use of toxic solvents or high temperatures (>60 °C) [80].

This extraction technique has been used as a method for valorizing oyster mushroom by-products. Meharwade et al. [76] studied the extraction with Protamex^®^-V8 of the stipe of *P. ostreatus*, with the aim of recovering small antioxidant peptides (1–3 kDa). However, the objective of this study was not to break down the cell wall of the mushroom, but to use endo- and exo-peptidases to induce enzymatic hydrolysis of structural proteins, generating bioactive peptides. Another study conducted on cuttings, leftovers, and mycelium from *P. ostreatus* observed the effect of different enzymes, such as protease or papain, on protein recovery [61]. This study showed a higher yield of EAE in the extraction capacity of these compounds compared to other technologies such as UAE or environmentally friendly aqueous extraction (EFAE).

Although the use of EAE in the extraction of proteins and peptides from mushroom by-products is a highly specific and efficient recovery strategy, the use of other hydrolytic enzymes could be of particular interest in the extraction of polysaccharides and β-glucans, compounds that are highly represented in the stipe, mycelium, and SMS of *P. ostreatus*. This approach has been used in other mushroom species, such as *Lentinus edodes* [81], where a study obtained good yields of polysaccharide extraction using enzymes such as cellulase, papain, and pectinase. Another study determined that treatment with α-amylase and amyloglucosidase was capable of promoting the extraction of α- and β-glucans in *Agaricus bisporus* [82]. This could be a good alternative strategy to valorize oyster mushroom by-products, as enzymes such as cellulases and hemicellulases could break down the β-1,4-glucosides of the cellulose/hemicellulose associated with the cell wall, which are especially present in by-products such as mycelium and fibrous remains of the stipe/SMS. Although EAE represents a promising strategy for the valorization of *P. ostreatus* by-products, this technique is highly dependent on the choice and cost of enzymes as well as optimal operating conditions (pH, temperature, reaction time). These factors make it necessary to optimize and combine with other sustainable extraction technologies to maximize yield and ensure economic viability.

### 3.5. Deep Eutectic Solvents (DES) Extraction

In recent years, deep eutectic solvent (DES) extraction has emerged as a sustainable alternative to conventional organic solvents, demonstrating high efficiency in the extraction of phenolic compounds, polysaccharides, and secondary metabolites from fungal matrices. DESs are mixtures of two or more compounds, including a hydrogen bond donor (HBD) and a hydrogen bond acceptor (HBA) (i.e., organic acids, sugars, or choline), whose interaction causes a decrease in the melting point of the mixture, resulting in a stable, biodegradable, non-volatile liquid with low toxicity [83]. Although no specific studies on the use of DES in *P. ostreatus* by-products have been documented yet, its use has been extended in recent years to different fungal species. Karabulut et al. (2024) [84] evaluated the effectiveness of DES in the extraction of proteins from agro-waste mushroom stems of *Agaricus bisporus*, demonstrating good protein retrieval and enhanced lignocellulosic disruption. DES has also been shown to be efficient in the extraction of polysaccharides. A study conducted on *Lentinus edodes* showed that the combination of choline chloride with hydrogen bond donors (i.e., urea, malonate, 1,2-propanediol) favored the extraction of polysaccharides and increased the antioxidant capacity of the extracts [85]. Finally, DES has been used for the extraction of various compounds of interest such as chitin [86], vitamin D [87], and ergosterol [88]. This growing evidence highlights the potential of applying DES to *P. ostreatus*, with the aim of recovering added-value compounds (polysaccharides, phenolic compounds) from by-products, contributing to a more circular and sustainable oyster mushroom production system.

### 3.6. Other Emerging Technologies and Comparative Perspective

Apart from the techniques already mentioned, other green extraction techniques may be of interest in the valorization of oyster mushrooms. Subcritical water extraction (SWE) is an extraction process that uses water in a subcritical state (120–200 °C, 3.5–20 MPa), which can break down cell walls and release polar compounds without the use of organic solvents [89]. This technology has been used in *P. ostreatus* SMS with the aim of extracting phenolic fractions and fermentable sugars [34]. In this study, SWE demonstrated higher yield and extraction efficiency compared to traditional methods and UAE, as well as better recovery of phenolic compounds and total carbohydrates. SWE can be a good alternative to the extraction of polar compounds such as polyphenols, sugars, and polysaccharides from wet matrices such as SMS or cuttings. However, this technique is not effective for the extraction of lipids or pure essential oils, which are better extracted with SFE or organic solvents. Furthermore, the use of high temperatures can cause the degradation of some sensitive compounds.

Another extraction technology of interest is pressurized liquid extraction (PLE), also known as accelerated solvent extraction (ASE). PLE uses solvents such as water and ethanol at high temperatures (50–200 °C) and pressure (10–15 MPa) to increase the solubility and diffusion of bioactive compounds. This approach has been validated in *P. ostreatus* as a powerful tool for extracting polysaccharides and β-glucans in the fruiting body, demanding lower solvent consumption and very short times compared to MAE [70]. On the other hand, although PLE is considered a green technique due to lower solvent consumption than classical methods, it still requires solvents (ethanol, methanol, hexane, acetone, etc.), which can generate potentially toxic residues.

Finally, other technologies such as pulsed electric fields (PEF) have been used in the extraction of certain compounds from *P. ostreatus*. For PEF treatment, high-intensity electric pulses (generally between 1 and 50 kV/cm) are applied for microseconds or milliseconds, generating an electric field that penetrates the cell membrane and causes electroporation [90]. Calleja-Gómez et al. [72] studied the effect of low-intensity PEF treatment (2.5 kV/cm) on oyster mushrooms, obtaining good results in the extraction of cinnamic acid, phenolic content, and antioxidant capacity measured by ORAC assay. A subsequent study with the same matrix under the same conditions demonstrated the ability of PEF treatment to recover carbohydrates, as well as a higher content of essential minerals such as Mg, Fe, P, and K [71]. These results reveal that PEF treatment can be a valuable strategy as a preliminary step in mushroom processing. However, the use of this technique has not been reported in *P. ostreatus* by-products, which could be of interest in reducing time and temperature in sensitive matrices (i.e., non-commercial pileus trimmings and by-products with high water content) prior to mild aqueous or hydroalcoholic extraction.

Overall, the performance of these green extraction technologies depends strongly on the polarity, stability, and structural location of the target compounds in *P. ostreatus* matrices. Among them, ultrasound-assisted extraction (UAE) and enzyme-assisted extraction (EAE) stand out for their mild conditions and versatility, showing high efficiency for recovering phenolics and polysaccharides, respectively. Microwave-assisted extraction (MAE) provides rapid heating and high yields, although excessive energy input may promote partial degradation of thermolabile compounds. Supercritical fluid extraction (SFE) and subcritical water extraction (SWE) allow selective recovery of non-polar and moderately polar compounds without toxic solvents, yet their scalability can be limited by equipment cost. Pulsed electric fields (PEF) mainly act as a non-thermal pretreatment that enhances mass transfer when combined with other techniques, while deep eutectic solvents (DES) show promise as next-generation, tunable media for extracting multiple compound classes under eco-friendly conditions.

Consequently, the selection of the optimal extraction strategy for *P. ostreatus* valorization should consider not only extraction yield but also the chemical nature of the desired compounds, operational costs, and potential integration into industrial processes. A comparative understanding of these technologies supports a rational and sustainable design of valorization pathways, linking the recovery of bioactive compounds with their potential incorporation into food products.

## 4. Valorization Through Functional Food Applications

### 4.1. Valorization of the Fruiting Body of P. ostreatus

In recent years, the food industry has shown growing interest in developing innovative formulations that not only provide additional health benefits but also help to modulate physiological functions. In this context, the use of by-products as functional ingredients represents a good strategy for promoting their valorization, while simultaneously enhancing the added value of new products. Specifically, by-products generated during the processing of *P. ostreatus* are a great source of bioactive compounds, including dietary fiber, β-glucans, ergosterol, polyphenols, and tocopherols. These compounds are associated with multiple health benefits, making their incorporation into functional foods a promising and commercially relevant approach.

#### 4.1.1. Meat Products

The fruiting body of oyster mushrooms has been used as a functional ingredient in a wide range of different products, with meat products being the most prevalent. Specifically, *P. ostreatus* has been used in the formulation of patties [91,92,93,94], sausages [95,96], pâté [97,98], meatballs [99,100], salami [101], and shredded meat [102]. Mushrooms have antimicrobial and antioxidant properties, which can improve the shelf life of meat products. However, their main use in these products is due to their high dietary fiber content, which is not present in animal products, as well as their potential use as a salt substitute [103]. *P. ostreatus* has a characteristic umami flavor that arises from the harmonious interaction of various components, such as certain amino acids, 5′-nucleotides, succinic acid, and umami peptides, making it ideal as a salt substitute without compromising sensory qualities [92,95].

#### 4.1.2. Bakery Products

The incorporation of *P. ostreatus* as an ingredient in bakery products has also been studied as a strategy to improve its functional properties. Some studies have reported success in enriching wheat and rye breads with oyster mushrooms [104,105,106,107,108,109] (Table 3), improving the nutritional composition and bioactive content without compromising dough handling or bread quality. Additionally, these studies have reported that oyster mushroom flour can increase the protein, polyphenol, and dietary fiber content of bread, while maintaining acceptable sensory and technological quality. In addition to bread, oyster mushrooms have also been tested as an ingredient in other products, such as cookies [110,111,112,113,114] or noodles [115], where their incorporation in the form of flour has demonstrated good sensory acceptance.

#### 4.1.3. Dairy Products

Furthermore, due to its rheological and functional properties, oyster mushrooms can also be a good alternative in the formulation of dairy products. In processed or reduced-fat cheeses, the β-glucans of *P. ostreatus* could act as hydrocolloids, helping to retain water and improve the cohesion of the protein matrix [116,117]. Furthermore, it has been shown that their incorporation into cheeses such as mozzarella or processed cheese can increase the total phenol content and antioxidant activity [118,119]. The inclusion of oyster mushrooms in yogurt formulations has also been studied [120,121,122]. Sakul et al. [121] studied the inclusion of 2–8% in yogurt, highlighting an improvement in the product’s antioxidant activity. In addition, the reformulation increased the total count of lactic acid bacteria and significantly promoted the survival of *Lactobacillus acidophilus*, which is essential for the development of a functional yogurt with probiotic properties.

### 4.2. Valorization of P. ostreatus By-Products

**Figure 3:**
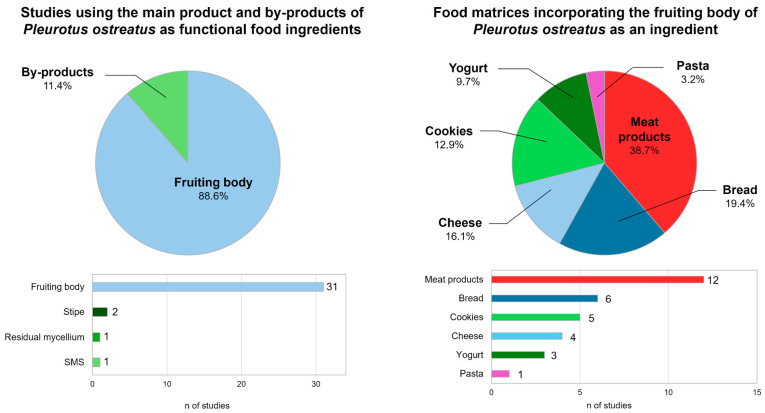
Studies on the use of *P. ostreatus* and its by-products as functional food ingredients and their application in different food matrices. Source: [91,92,93,94,95,96,97,98,99,100,101,102,104,105,106,107,108,109,110,111,112,113,114,115,116,117,118,119,120,121,122,123,124,125,126].

Although the benefits of incorporating the fruiting body of *P. ostreatus* are supported by more than 30 studies, there is very limited existing literature on the use of oyster mushroom by-products in the formulation of functional foods (Figure 3). Specifically, residual mycelium from *P. ostreatus* has been used in the production of Karish cheese [123]. In this study, the incorporation of these by-products showed a decrease in coagulation time, as well as an improvement in rheological characteristics in fresh cheese samples and during storage. Sensory evaluation showed reformulated cheese was more acceptable to panelists than cheeses made with ferment or rennet. Spent mushroom substrate has also been used in food formulation. Moshtaghian et al. [124] studied the effect of incorporating SMS into bread made from 50% crop residues and 50% wheat flour. This by-product provided the bread with a high content of niacin, fiber, and β-glucan, but the new formulation scored higher in terms of darkness, dryness, sour taste, and bitter aftertaste in the sensory assessment. The use of stipe as a functional ingredient has been studied more extensively. A recent study [125] investigated the use of *P. ostreatus* stipe as a partial substitute for starch and sodium in *mortadella*. The incorporation of stipe flour preserved texture, color, and aroma, but revealed significantly lower overall acceptability in sensory analysis. The new formulation achieved a similar perception of salinity, indicating the potential of oyster mushroom by-products as natural flavor enhancers. In addition, El-Maaty et al. [126] studied the impact of replacing 5, 10, and 15% of wheat flour with mushroom stipe powder in bread. The reformulations significantly increased the crude fiber content of the bread. In addition, the baking properties, color, and sensory evaluation showed that wheat flour could be replaced by 5 or 10% mushroom by-product to produce good-quality bread. Overall, most research on *P. ostreatus* as a functional ingredient has focused on the fruiting body (Figure 3), while by-products have received much less attention despite their nutritional benefits. This asymmetry highlights the need to promote studies specifically aimed at revaluing by-products in the formulation of food products.

From an applied perspective, the compositional diversity of each by-product opens up a range of possibilities for the design of new foods. The stipe is a good source of dietary fiber and β-glucans, so it could be used as a structuring ingredient in meat products or vegetable analogues, improving texture and providing prebiotic properties. Furthermore, due to its high glucan and polysaccharide content, the stipe has rheological properties with great potential. In fact, studies have shown that after aqueous extraction of this by-product, gel-like polysaccharides with bioactive properties can be obtained [127], which could have applications in the food industry. On the other hand, residual mycelium is an excellent source of dietary fiber, especially SDF, which could be used as a potential ingredient in functional beverages (smoothies, dairy products, vegetable drinks, fiber-enriched water), since soluble fiber disperses well and does not have the grain-like texture of insoluble fiber. In addition, it could also be used in baking and pastry making, as these nutrients can improve texture and increase moisture retention. Although SMS has gained interest due to its possible use in animal feed [128], it also holds promise as a functional ingredient for humans due to its high content of dietary fiber and polyphenols. Nevertheless, the potential use of *P. ostreatus* by-products in the food industry still has certain limitations, and further studies are needed to confirm their safety and applicability [129,130,131].

**Table 3:** Valorization of *P. ostreatus* and its by-products as functional ingredients in different food matrices.

Botanical Part	Food Matrix	Functional Role	Key Outcomes	Ref.
Fruiting Body	Pâté	Partial replacement of fat and salt	Enhancement in moisture, dietary fiber, and protein	[97]
			Acceptable sensory parameters	
Fruiting Body	Beef Burgers	Salt replacement	Higher lipid oxidation	[92]
			No impact on sensory parameters	
Fruiting Body	Beef meatballs	Partial replacement of beef	Increased fiber and decreased fat	[99]
Stipe	*Mortadella*	Sodium and starch replacement	Improved protein content and a significant reduction in residual nitrite levels Lower sensory acceptability	[125]
Fruiting Body	Rye Bread	Replacement of rye flour	Higher antioxidant activity Prolonged starch gelatinization time and increased xylolytic activity	[108]
SMS	Wheat Bread	Partial wheat flour replacement	Higher content of niacin, fiber, and β-glucan Negative impact on color and sensory results	[124]
Stipe	Wheat Bread	Partial wheat flour replacement	Increase in protein and fiber content Acceptable color and sensory parameters under 5% replacement	[126]
Fruiting Body	Cookies	Functional fortification	Enhanced protein, fiber, vitamin D, and folic acid content Higher antioxidant activity	[112]
Fruiting Body	Yogurt	Incorporation as a prebiotic	Higher viability of *Lactobacillus acidophilus* bacteria and lactic acid bacteria	[121]
			Enhanced antioxidant activity	
Fruiting Body	Mozzarella cheese	Functional fortification	Improved texture and water-holding capacity Higher content of phenolic compounds	[119]
Mycelium	Karish cheese	Rennet replacement	Decrease in coagulation time Improved sensory acceptability	[123]

Although most studies have focused on the incorporation of *P. ostreatus* by-products in the form of dried powders, the use of extracts obtained through green extraction technologies (such as UAE, MAE, EAE, or DES) offers significant advantages from both technological and nutritional perspectives. Extracts allow a more standardized and concentrated recovery of bioactive compounds, improving their homogeneity, solubility, and bioavailability within food matrices. This results in enhanced functional effects, such as antioxidant or prebiotic activity, without the negative sensory impact often associated with high levels of by-product powder incorporation (i.e., darker color, increased dryness, or bitterness). Moreover, extracts can be more easily encapsulated, emulsified, or integrated into food systems as liquid or semi-solid ingredients, facilitating product formulation and stability. Therefore, combining extraction and incorporation strategies could maximize the technological and health potential of *P. ostreatus* by-products, advancing toward a more efficient and sustainable valorization pathway.

## 5. Future Challenges and Opportunities

Although green extraction technologies are an excellent way to valorize oyster mushrooms through the extraction of bioactive compounds of interest, such as β-glucans, dietary fiber, or polyphenols, some limitations must be considered for their industrial-scale application. Overall, studies using these extraction technologies have mostly been performed on a laboratory scale, which limits their direct translation to industrial processes, where controlling things like temperature, pressure, or pH is more complex and costly. Furthermore, although technologies such as SFE and SWE show clear advantages in eliminating the use of toxic solvents, they require high energy consumption and specialized equipment to be scalable. Similarly, techniques such as MAE and UAE offer moderate yields and results similar to conventional methods; however, they often require high temperatures, which can lead to the degradation of thermolabile compounds. Additionally, their efficiency depends largely on the type of by-product and the target compound. Likewise, EAE is selective and can be effective in the extraction of polysaccharides or proteins, but its potential industrial use depends on the type and cost of enzymes, which limits its viability. These limitations in the use of green extraction in oyster mushrooms make it necessary to conduct research into pilot studies that validate their actual scalability. In addition, it is necessary to evaluate technological synergies that offset the individual limitations of each technique. Combinations such as PEF + UAE could improve extraction efficiency, since PEF facilitates cell permeabilization and UAE accelerates the diffusion of compounds, allowing for better yields with less energy and solvent use.

Nevertheless, the extraction of bioactive compounds using these extractive technologies is necessary for the proper valorization of by-products, especially in their application as functional ingredients. For example, by-products such as SMS must be extracted prior to incorporation due to their rheological characteristics. It should be noted that there are currently very few studies supporting the use of *P. ostreatus* by-products as a functional ingredient; however, there is extensive literature on their nutritional benefits and the possibility of extraction using green technologies. For this reason, further research is needed to ensure that the resulting ingredients meet the requirements for safe and commercially viable use in food systems. Sensory acceptance plays a key role in the commercialization of new ingredients. The direct addition of unprocessed by-products can negatively affect flavor, color, or texture, reducing consumer acceptance. In addition, by-products such as SMS may contain residues of microorganisms, pesticides, heavy metals, or contaminants from the original substrate, requiring additional treatments to ensure their safety before incorporation into food. In addition, the digestibility of extracts from these by-products needs to be evaluated, as a large part of the fiber is insoluble and lignified, which may limit their inclusion at high levels and compromise their functional properties if not properly processed.

In addition, the regulatory framework for incorporating ingredients into food varies by region, being governed in Europe by Regulation (EU) 2015/2283 on Novel Foods and in the United States by the generally recognized as safe (GRAS) notification process. Although mushrooms and their components are considered safe when cultivated under controlled conditions, by-products such as stipe, mycelium, and SMS are not automatically covered by this status. Therefore, additional toxicological, allergenic, and microbiological evaluations are required to demonstrate their safety and absence of contaminants such as heavy metals or residues. Further research into safe use and the provision of supporting toxicological data would facilitate its recognition within existing regulatory frameworks, ultimately allowing the incorporation of *P. ostreatus* by-products as safe, functional, and sustainable food ingredients.

## 6. Conclusions

The by-products of *P. ostreatus* (stipe, residual mycelium, and SMS) are rich in compounds of high nutritional and functional interest, such as dietary fiber, β-glucans, polyphenols, and essential minerals. However, their potential has been largely unexploited, with most being used for low-value-added purposes or directly discarded, representing a loss of valuable resources.

This review has highlighted green extraction technologies as potential strategies for revalorizing these by-products. Techniques such as ultrasound-assisted extraction (UAE), microwave-assisted extraction (MAE), supercritical fluid extraction (SFE), subcritical water extraction (SWE), enzyme-assisted extraction (EAE), and pulsed electric fields (PEF) application are emerging as sustainable alternatives to conventional methods and have shown promising results in the recovery of bioactive compounds from oyster mushroom by-products. Although these techniques allow bioactive compounds to be recovered without the use of harmful solvents, contributing to the circular economy, there are still challenges in their industrial scalability. Many investigations are limited to the laboratory scale and have limitations in terms of cost, energy efficiency, and stability of the extracted compounds. Moreover, the application of these by-products in the formulation of functional foods shows great economic and sustainable potential. The incorporation of the fruiting body of *P. ostreatus* has shown promising outcomes in replacing salt in meat products, as a functional hydrocolloid in dairy products, and as a fortification of phenolic compounds and β-glucans in bakery products. However, although the agro-industrial by-products of *P. ostreatus* have similar nutritional qualities to those of the main product, applied research into the incorporation of these by-products is very limited, which represents a research gap with great potential for application in the valorization of biowaste. Overall, the sustainable valorization of oyster mushrooms can not only reduce waste and optimize resources but also open up new avenues for food innovation. For this reason, further research is needed to validate these technologies under pilot and industrial conditions, as well as to evaluate the sensory acceptance and microbiological safety of potential functional ingredients.

## Data Availability

No new data were created or analyzed in this study. Data sharing is not applicable to this article.

## Abbreviations

The following abbreviations are used in this manuscript:

ABTS	2,2′-azino-bis(3-ethylbenzothiazoline-6-sulfonic acid)
BCAA	Branched-Chain Amino Acids
Ca	Calcium
Cr	Chromium
Cu	Copper
DES	Deep Eutectic Solvents
DW	Dry Weight
DPPH	2,2-diphenyl-1-picrylhydrazyl
Fe	Iron
FRAP	Ferric Reducing Antioxidant Power
FW	Fresh Weight
GAE	Gallic Acid Equivalents
GRAS	Generally Recognized As Safe
IDF	Insoluble Dietary Fiber
K	Potassium
Mg	Magnesium
Mn	Manganese
Mo	Molybdenum
MUFA	Monounsaturated Fatty Acids
Na	Sodium
nd	Not Detected
ORAC	Oxygen Radical Absorbance Capacity
P	Phosphorus
PUFA	Polyunsaturated Fatty Acids
ROS	Reactive Oxygen Species
SDF	Soluble Dietary Fiber
SFE	Supercritical Fluid Extraction
SMS	Spent Mushroom Substrate
SWE	Subcritical Water Extraction
TDF	Total Dietary Fiber
TE	Trolox Equivalents
TPC	Total Phenolic Content
UAE	Ultrasound-Assisted Extraction
MAE	Microwave-Assisted Extraction
PEF	Pulsed Electric Fields
SDGs	Sustainable Development Goals
Zn	Zinc

## References

[1] Effiong M.E., Umeokwochi C.P., Afolabi I.S., Chinedu S.N. (2023). Assessing the Nutritional Quality of *Pleurotus ostreatus* (Oyster Mushroom). Front. Nutr..

[2] Panagiota D., Katerina F., Maria M.E., Marianna D., Ilias D., Chrysavgi G., Seraphim P. (2023). Examining the Impact of Substrate Composition on the Biochemical Properties and Antioxidant Activity of *Pleurotus* and *Agaricus* Mushrooms. Fermentation.

[3] Effiong M.E., Umeokwochi C.P., Afolabi I.S., Chinedu S.N. (2024). Comparative Antioxidant Activity and Phytochemical Content of Five Extracts of *Pleurotus ostreatus* (Oyster Mushroom). Sci. Rep..

[4] Dündar A., Yalçın P., Arslan N., Acay H., Hatipoğlu A., Boğa M., Karahan S., Yaprak B. (2024). Effect of *Pleurotus ostreatus* Water Extract Consumption on Blood Parameters and Cytokine Values in Healthy Volunteers. J. Am. Nutr. Assoc..

[5] Iqbal T., Sohaib M., Iqbal S., Rehman H. (2024). Exploring Therapeutic Potential of *Pleurotus ostreatus* and *Agaricus bisporus* Mushrooms against Hyperlipidemia and Oxidative Stress Using Animal Model. Foods.

[6] Carminati G., Di Foggia M., Garagozzo L., Di Francesco A. (2024). Mushroom By-Products as a Source of Growth Stimulation and Biochemical Composition Added-Value of *Pleurotus ostreatus*, *Cyclocybe cylindracea*, and *Lentinula edodes*. Foods.

[7] Torres-Martínez B.D.M., Vargas-Sánchez R.D., Torrescano-Urrutia G.R., Esqueda M., Rodríguez-Carpena J.G., Fernández-López J., Perez-Alvarez J.A., Sánchez-Escalante A. (2022). *Pleurotus* Genus as a Potential Ingredient for Meat Products. Foods.

[8] Atallah E., Zeaiter J., Ahmad M.N., Leahy J.J., Kwapinski W. (2021). Hydrothermal Carbonization of Spent Mushroom Compost Waste Compared against Torrefaction and Pyrolysis. Fuel Process. Technol..

[9] Calleja-Gómez M., Roig P., Pateiro M., Domínguez-Valencia R., Lorenzo J.M., Fernández-López J., Viuda-Martos M., Pérez-Álvarez J., Martínez-Zamora L., Nieto G. (2024). Health-Promoting Benefits of Plant-Based by-Product Extracts Obtained by Innovative Technologies. Curr. Opin. Food Sci..

[10] Imamoglu E. (2024). Green Extraction Processes from Renewable Biomass to Sustainable Bioproducts. Bioresour. Technol. Rep..

[11] Ospina-Maldonado S., Martin-Gómez H., Cardoso-Ugarte G.A. (2024). From Waste to Wellness: A Review on the Harness of Food Industry by-Products for Sustainable Functional Food Production. Int. J. Food Sci. Technol..

[12] Lesa K.N., Khandaker M.U., Mohammad Rashed Iqbal F., Sharma R., Islam F., Mitra S., Emran T. (2022). Bin Nutritional Value, Medicinal Importance, and Health-Promoting Effects of Dietary Mushroom (*Pleurotus ostreatus*). J. Food Qual..

[13] Bach F., Helm C.V., Bellettini M.B., Maciel G.M., Haminiuk C.W.I. (2017). Edible Mushrooms: A Potential Source of Essential Amino Acids, Glucans and Minerals. Int. J. Food Sci. Technol..

[14] Al Faruq A., Farahnaky A., Torley P.J., Buckow R., Eri R., Majzoobi M. (2025). Sustainable Approaches to Boost Soluble Dietary Fibre in Foods: A Path to Healthier Foods. Food Hydrocoll..

[15] Majesty D.K.C., Winner K., Univeristy R., Prince O., Ijeoma E. (2019). Nutritional, Anti-Nutritional and Biochemical Studies on the Oyster Mushroom, *Pleurotus ostreatus*. EC Nutr..

[16] Singh R.P., Bhardwaj A. (2023). β-Glucans: A Potential Source for Maintaining Gut Microbiota and the Immune System. Front. Nutr..

[17] Swain J.H., Minisola S., Razzaque M.S., Wimalawansa S.J. (2025). Minerals and Human Health: From Deficiency to Toxicity. Nutrients.

[18] Fazlić D., Udovičić A., Valjevac M., Hećo M., Karić L., Sinanović Ć.Z., Murtić S. (2024). Comparative Study of the Mineral Composition of Selected Cultivated Mushrooms. J. Appl. Hortic..

[19] Raman J., Jang K.Y., Oh Y.L., Oh M., Im J.H., Lakshmanan H., Sabaratnam V. (2020). Cultivation and Nutritional Value of Prominent *Pleurotus* spp.: An Overview. Mycobiology.

[20] Igile G.O., Bassey S.O., Essien N. (2020). Nutrient composition of oyster mushroom (*Pleurotus ostreatus*), grown on rubber wood sawdust in Calabar, Nigeria, and the nutrient variability between harvest times. Eur. J. Food Sci. Technol..

[21] Tian H., Li Y.F., Jiao G.L., Sun W.Y., He R.R. (2024). Unveiling the Antioxidant Superiority of α-Tocopherol: Implications for Vitamin E Nomenclature and Classification. Free Radic. Biol. Med..

[22] Palacios I., Lozano M., Moro C., D’Arrigo M., Rostagno M.A., Martínez J.A., García-Lafuente A., Guillamón E., Villares A. (2011). Antioxidant Properties of Phenolic Compounds Occurring in Edible Mushrooms. Food Chem..

[23] Koutrotsios G., Kalogeropoulos N., Stathopoulos P., Kaliora A.C., Zervakis G.I. (2017). Bioactive Compounds and Antioxidant Activity Exhibit High Intraspecific Variability in *Pleurotus ostreatus* Mushrooms and Correlate Well with Cultivation Performance Parameters. World J. Microbiol. Biotechnol..

[24] Jayakumar T., Thomas P.A., Geraldine P. (2009). In-Vitro Antioxidant Activities of an Ethanolic Extract of the Oyster Mushroom, *Pleurotus ostreatus*. Innov. Food Sci. Emerg. Technol..

[25] Bermúdez-Gómez P., Fernández-López J., Pérez-Clavijo M., Viuda-Martos M. (2024). Optimization of Mushroom (*Agaricus bisporus* and *Pleurotus ostreatus*) By-Products Processing for Prospective Functional Flour Development. Foods.

[26] Golian M., Chlebová Z., Žiarovská J., Benzová L., Urbanová L., Hovaňáková L., Chlebo P., Urminská D. (2022). Analysis of Biochemical and Genetic Variability of *Pleurotus ostreatus* Based on the β-Glucans and CDDP Markers. J. Fungi.

[27] Pérez-Bassart Z., Falcó I., Martínez-Sanz M., Martínez-Abad A., Sánchez G., López-Rubio A., Fabra M.J. (2024). Antiviral and Technological Properties of β-Glucan-Rich Aqueous Fractions from *Pleurotus ostreatus* Waste Biomass. Food Hydrocoll..

[28] Szewczyk K., Chojnacka A., Górnicka M. (2021). Tocopherols and Tocotrienols—Bioactive Dietary Compounds; What Is Certain, What Is Doubt?. Int. J. Mol. Sci..

[29] Zawadzka A., Janczewska A., Kobus-Cisowska J., DziedzińskiID M., Siwulski M., Czarniecka-SkubinaID E., Stuper-Szablewska K. (2022). The Effect of Light Conditions on the Content of Selected Active Ingredients in Anatomical Parts of the Oyster Mushroom (*Pleurotus ostreatus* L.). PLoS ONE.

[30] Cherno N., Osollina S., Nikitina A. (2016). Chemical Composition of *Agaricus bisporus* and *Pleurotus ostreatus* Fruiting Bodies and Their Morphological Parts. Food Environ. Saf. J..

[31] Silva M., Ramos A.C., Lidon F.J., Reboredo F.H., Gonçalves E.M. (2024). Pre- and Postharvest Strategies for *Pleurotus ostreatus* Mushroom in a Circular Economy Approach. Foods.

[32] Soltan M.A., Shewita R.S., Matroud O.A., Alkeridis L.A., Sayed S., Shukry M., El-Shobokshy S.A. (2024). Lignocellulose and Probiotic Supplementation in Broiler Chicken Diet: Effect on Growth Performance, Digestive Health, Litter Quality, and Genes Expression. Poult. Sci..

[33] Ahmad Zakil F., Mohd Isa R., Mohd Sueb M.S., Isha R. (2022). Growth Performance and Mineral Analysis of *Pleurotus ostreatus* (Oyster Mushroom) Cultivated on Spent Mushroom Medium Mixed with Rubber Tree Sawdust. Mater. Today Proc..

[34] Klausen S.J., Falck-Ytter A.B., Strætkvern K.O., Martin C. (2023). Evaluation of the Extraction of Bioactive Compounds and the Saccharification of Cellulose as a Route for the Valorization of Spent Mushroom Substrate. Molecules.

[35] He J., Qiu Y., Ji X., Liu X., Qiu Z., Xu J., Xia J. (2021). A Novel Strategy for Producing Cellulase from Trichoderma Reesei with Ultrasound-assisted Fermentation Using Spent Mushroom Substrate. Process Biochem..

[36] Laforteza J.C., Reyes R.G., Trinidad T.P. (2020). Dietary Fiber Contents and Its Fermentability In Vitro of *Pleurotus ostreatus* Cv. Florida Mycelia (Agaricomycetes). Int. J. Med. Mushrooms.

[37] Törős G., El-Ramady H., Béni Á., Peles F., Gulyás G., Czeglédi L., Rai M., Prokisch J. (2024). *Pleurotus ostreatus* Mushroom: A Promising Feed Supplement in Poultry Farming. Agriculture.

[38] Thalita Ferreira Silva N., Reis Venancio A., Tokuda Martos E., Clara Gomes Oliveira A., Alice Andrade Oliveira A., da Silva Mutz Y., Antonio Nunes C., Lucía Mondragón-Bernal O., Guilherme Lembi Ferreira Alves J. (2024). Fish Fillet Analogue Using Formulation Based on Mushroom (*Pleurotus ostreatus*) and Enzymatic Treatment: Texture, Sensory, Aromatic Profile and Physicochemical Characterization. Foods.

[39] Alam N., Amin R., Khan A., Ara I., Shim M.J., Lee M.W., Lee T.S. (2008). Nutritional Analysis of Cultivated Mushrooms in Bangladesh—*Pleurotus ostreatus*, *Pleurotus sajor-caju*, *Pleurotus florida* and *Calocybe indica*. Mycobiology.

[40] Naim L., Alsanad M.A., Shaban N., El Sebaaly Z., Abou Fayssal S., Sassine Y.N. (2020). Production and Composition of *Pleurotus ostreatus* Cultivated on Lithovit^®^-Amino25 Supplemented Spent Substrate. AMB Express.

[41] Sopanrao P.S., Abrar A.S., Manoharrao T.S., Vaseem B.M.M. (2010). Nutritional Value of *Pleurotus ostreatus* (Jacq:Fr) Kumm Cultivated on Different Lignocellulosic Agro-Wastes. Innov. Rom. Food Biotechnol..

[42] Irshad A., Tahir A., Sharif S., Khalid A., Ali S., Naz A., Sadia H., Ameen A. (2023). Determination of Nutritional and Biochemical Composition of Selected *Pleurotus* spps. Biomed. Res. Int..

[43] Synytsya A., Míčková K., Jablonský I., Sluková M., Čopíková J. (2008). Mushrooms of Genus *Pleurotus* as a Source of Dietary Fibres and Glucans for Food Supplements. Czech J. Food Sci..

[44] Frioui M., Yimer G.A., Shamtsyan M., Barakova N., Dozortseva A., Kolesnikov B., Sadovoy V., Kiprushkina E. (2024). Isolation of Bioactive Beta-Glucans from Mycelium of *Pleurotus ostreatus* Mushroom. Bioact. Compd. Health Dis..

[45] Pérez-Bassart Z., Fabra M.J., Martínez-Abad A., López-Rubio A. (2023). Compositional Differences of β-Glucan-Rich Extracts from Three Relevant Mushrooms Obtained through a Sequential Extraction Protocol. Food Chem..

[46] Papaspyridi L.M., Katapodis P., Gonou-Zagou Z., Kapsanaki-Gotsi E., Christakopoulos P. (2010). Optimization of Biomass Production with Enhanced Glucan and Dietary Fibres Content by *Pleurotus ostreatus* ATHUM 4438 under Submerged Culture. Biochem. Eng. J..

[47] Tagkouli D., Kaliora A., Bekiaris G., Koutrotsios G., Christea M., Zervakis G.I., Kalogeropoulos N. (2020). Free Amino Acids in Three Pleurotus Species Cultivated on Agricultural and Agro-Industrial By-Products. Molecules.

[48] Vetter J., Hajdú C., Gyorfi J., Maszlavér P. (2005). Mineral Composition of the Cultivated Mushrooms *Agaricus bisporus*, *Pleurotus ostreatus* and *Lentinula edodes*. Acta Aliment..

[49] Jin Z., Li Y., Ren J., Qin N. (2018). Yield, Nutritional Content, and Antioxidant Activity of *Pleurotus ostreatus* on Corncobs Supplemented with Herb Residues. Mycobiology.

[50] Fontes Vieira P.A., Gontijo D.C., Vieira B.C., Fontes E.A.F., Assunção L.S.d., Leite J.P.V., Oliveira M.G.d.A., Kasuya M.C.M. (2013). Antioxidant Activities, Total Phenolics and Metal Contents in *Pleurotus ostreatus* Mushrooms Enriched with Iron, Zinc or Lithium. LWT—Food Sci. Technol..

[51] Purnomo A.S., Sariwati A., Fatmawati S., Puspitasari F.E. (2023). Effect of the Coconut Coir (*Cocos nucifera*) as a Growth Medium for *Pleurotus ostreatus* (Oyster Mushroom) on Mineral and Vitamin B Contents. Hayati.

[52] Zhang W., Liu Q., Zhou T., Chen N., Jiang W. (2024). Aqueous Extraction of Ergothioneine from Mycelia of *Pleurotus ostreatus* and Ergothioneine Accumulation Regularity during Submerged Fermentation. J. Appl. Hortic..

[53] Tian X., Thorne J.L., Moore J.B. (2023). Ergothioneine: An Underrecognised Dietary Micronutrient Required for Healthy Ageing?. Br. J. Nutr..

[54] Vásquez L.S., Sopo V., Suesca-Díaz A., Morales-Fonseca D. (2023). Elaboration of a Biomaterial from *Pleurotus ostreatus* Mycelium and Residual Biomass, as an Alternative to Synthetic Materials. Chem. Eng. Trans..

[55] Saini R., Kaur G., Brar S.K. (2024). Textile Residue-Based Mycelium Biocomposites from *Pleurotus ostreatus*. Mycology.

[56] Kumar K., Srivastav S., Sharanagat V.S. (2021). Ultrasound Assisted Extraction (UAE) of Bioactive Compounds from Fruit and Vegetable Processing by-Products: A Review. Ultrason. Sonochem..

[57] Bezerra F.d.S., Koblitz M.G.B. (2025). Extraction of Phenolic Compounds from Agro-Industrial By-Products Using Natural Deep Eutectic Solvents: A Review of Green and Advanced Techniques. Separations.

[58] Kuvendziev S., Lisichkov K., Marinkovski M., Stojchevski M., Dimitrovski D., Andonovikj V. (2024). Valorization of Tomato Processing By-Products: Predictive Modeling and Optimization for Ultrasound-Assisted Lycopene Extraction. Ultrason. Sonochem..

[59] Macias-Frotto B., Rostro-Alanís M., Escobedo-Avellaneda Z., Welti-Chanes J. (2024). Conventional and Innovative Methods for Pectin Extraction from Agro-Industrial By-Products. Food Eng. Rev..

[60] Estefes-Duarte J.A., Estefes-Duarte M.O., Pérez-Soto E., Jiménez-Alvarado R., Hernández-Soto I., Ceno-bio-Galindo A.d.J. (2025). Optimization of the Extraction of Antioxidant Compounds from the Depleted Substrate of *Pleurotus ostreatus* Using Ultrasound as a Valorization Alternative. Rev. Gestão—RGSA.

[61] Prandi B., Cigognini I.M., Faccini A., Zurlini C., Rodríguez Ó., Tedeschi T. (2023). Comparative Study of Different Protein Extraction Technologies Applied on Mushrooms By-Products. Food Bioproc. Tech..

[62] Dai Y., Wang L., Chen X., Song A., He L., Wang L., Huang D. (2023). *Lentinula edodes* Sing Polysaccharide: Extraction, Characterization, Bioactivities, and Emulsifying Applications. Foods.

[63] Wang Q., Cheng J., Wang L., Yan S., Wang R., Zhang H., Shao H., Yang X. (2018). Valorization of Spent Shiitake Substrate for Recovery of Antitumor Fungal Sterols by Ultrasound-Assisted Extraction. J. Food Biochem..

[64] Umaña M., Eim V., Garau C., Rosselló C., Simal S. (2020). Ultrasound-Assisted Extraction of Ergosterol and Antioxidant Components from Mushroom by-Products and the Attainment of a β-Glucan Rich Residue. Food Chem..

[65] Vezaro F.D., da Rosa B.V., Kuhn R.C. (2022). Ultrasound-Assisted Extraction of β-Glucans from *Lentinula edodes* Using Natural Deep Eutectic Solvent and Water. J. Chem. Technol. Biotechnol..

[66] Solaberrieta I., Mellinas C., Jiménez A., Garrigós M.C. (2022). Recovery of Antioxidants from Tomato Seed Industrial Wastes by Microwave-Assisted and Ultrasound-Assisted Extraction. Foods.

[67] de la Fuente B., Pinela J., Mandim F., Heleno S.A., Ferreira I.C.F.R., Barba F.J., Berrada H., Caleja C., Barros L. (2022). Nutritional and Bioactive Oils from Salmon (*Salmo Salar*) Side Streams Obtained by Soxhlet and Optimized Mi-crowave-Assisted Extraction. Food Chem..

[68] Davis E.J., Spadoni Andreani E., Karboune S. (2021). Production of Extracts Composed of Pectic Oligo/Polysaccharides and Polyphenolic Compounds from Cranberry Pomace by Microwave-Assisted Extraction Process. Food Bioproc. Tech..

[69] Harun M.U., Palma M., Setyaningsih W. (2025). Development and Validation of Microwave-Assisted Extraction for Phenolic Compound Profiling in Diverse Oyster Mushrooms (*Pleurotus* spp.) Sourced from Various Geographical Regions. J. Agric. Food Res..

[70] Smiderle F.R., Morales D., Gil-Ramírez A., de Jesus L.I., Gilbert-López B., Iacomini M., Soler-Rivas C. (2017). Evaluation of Microwave-Assisted and Pressurized Liquid Extractions to Obtain β-d-Glucans from Mushrooms. Carbohydr. Polym..

[71] Calleja-Gómez M., Martí-Quijal F.J., Roig P., Castagnini J.M., Barba F.J. (2025). Pulsed Electric Field Extracts Obtained from Edible Mushrooms: A Detailed ICP-MS Analysis of Their Mineral and Heavy Metal Contents and Their Cytotoxic Effect on CACO-2 Cells. Food Bioproc. Tech..

[72] Calleja-Gómez M., Roig P., Rimac Brnčić S., Barba F.J., Castagnini J.M. (2023). Scanning Electron Microscopy and Triple TOF-LC-MS-MS Analysis of Polyphenols from PEF-Treated Edible Mushrooms *(L. edodes*, *A. brunnescens*, and *P. ostreatus*). Antioxidants.

[73] Bhattacharya M., Srivastav P.P., Mishra H.N. (2014). Optimization of Process Variables for Supercritical Fluid Extraction of Ergothioneine and Polyphenols from *Pleurotus ostreatus* and Correlation to Free-Radical Scavenging Activity. J. Supercrit. Fluids.

[74] Barbosa J.R., Maurício M.M., Oliveira L.C., Luiza L.H., Almada-Vilhena A.O., Oliveira R.M., Pieczarka J.C., Brasil D.d.S.B., Carvalho Junior R.N. (2020). Obtaining Extracts Rich in Antioxidant Polysaccharides from the Edible Mushroom *Pleurotus ostreatus* Using Binary System with Hot Water and Supercritical CO_2_. Food Chem..

[75] Krivošija S., Nastić N., Karadžić Banjac M., Kovačević S., Podunavac-Kuzmanović S., Vidović S. (2025). Supercritical Extraction and Compound Profiling of Diverse Edible Mushroom Species. Foods.

[76] Meharwade S. (2020). Oyster Mushroom (*Pleurotus ostreatus*) Stipe Peptides as In-Vitro Radical Scavenging, Ferrous Iron Chelating, & Ferric Reducing Antioxidant Compounds. Master’s Thesis.

[77] Navarro-Simarro P., Gómez-Gómez L., Rubio-Moraga Á., Moreno-Gimenez E., López-Jimenez A., Prieto A., Ahrazem O. (2025). Valorization of Mushroom By-Products for Sustainability: Exploring Antioxidant and Prebiotic Properties. J. Food Biochem..

[78] Zhang J., Wu H. (2025). Valorization of Bioactive Compounds from Food By-Products Using Supercritical Fluid Extraction: A Technological and Industrial Perspective. Food Chem..

[79] Rangsinth P., Sharika R., Pattarachotanant N., Duangjan C., Wongwan C., Sillapachaiyaporn C., Nilkhet S., Wongsirojkul N., Prasansuklab A., Tencomnao T. (2023). Potential Beneficial Effects and Pharmacological Prop-erties of Ergosterol, a Common Bioactive Compound in Edible Mushrooms. Foods.

[80] Wani K.M., Nayana P., Wani K.M., Wani K.M. (2024). Unlocking the Green Potential: Sustainable Extraction of Bioactives from Orange Peel Waste for Environmental and Health Benefits. J. Food Meas. Charact..

[81] Zhao Y., Wang J., Wu Z., Yang J., Li W., Shen L. (2016). Extraction, Purification and Anti-Proliferative Activities of Polysaccharides from *Lentinus edodes*. Int. J. Biol. Macromol..

[82] You S.W., Šimora V., Ivanišová E., Jančo I., Chlebová Z., Ďúranová H., Gabríny L., Kačániová M., de Medeiros F.G.M., Hoskin R.T. (2025). Bioactive and Antioxidant Potential *Agaricus bisporus* Extracts Obtained by Different Extraction Methods and UV-B Irradiation. Food Bioeng..

[83] Hansen B.B., Spittle S., Chen B., Poe D., Zhang Y., Klein J.M., Horton A., Adhikari L., Zelovich T., Doherty B.W. (2020). Deep Eutectic Solvents: A Review of Fundamentals and Applications. Chem. Rev..

[84] Karabulut G., Köroğlu D.G., Feng H., Karabulut Z. (2024). Sustainable Fungi-Based Protein Extraction from Agro-Waste Mushroom Stem Using Deep Eutectic Solvents. Food Chem. X.

[85] Zhang J., Ye Z., Liu G., Liang L., Wen C., Liu X., Li Y., Ji T., Liu D., Ren J. (2022). Subcritical Water Enhanced with Deep Eutectic Solvent for Extracting Polysaccharides from *Lentinus edodes* and Their Antioxidant Activities. Molecules.

[86] Ozel N., Elibol M. (2024). Chitin and Chitosan from Mushroom (*Agaricus bisporus*) Using Deep Eutectic Solvents. Int. J. Biol. Macromol..

[87] Patil J., Ghodke S., Jain R., Dandekar P. (2018). Extraction of Vitamin D from Button Mushroom (*Agaricus bisporus*) Using Deep Eutectic Solvent and Ultrasonication. ACS Sustain. Chem. Eng..

[88] Liu Z., Deng M., Qu Y., Liang N., Zhao L. (2023). An Efficient Extraction Method for Ergosterol from *Lentinus edodes* Stem by Ultrasonic-Assisted Natural Deep Eutectic Solvent. Microchem. J..

[89] Majeed T., Shabir I., Srivastava S., Maqbool N., Dar A.H., Jan K., Pandey V.K., Shams R., Bashir I., Dash K.K. (2024). Valorization of Food Wastes by Implementation of Subcritical Water Extraction: A Comprehensive Review. Trends Food Sci. Technol..

[90] Chatzimitakos T., Athanasiadis V., Kalompatsios D., Mantiniotou M., Bozinou E., Lalas S.I. (2023). Pulsed Electric Field Applications for the Extraction of Bioactive Compounds from Food Waste and By-Products: A Critical Review. Biomass.

[91] Torres-Martínez B.d.M., Vargas-Sánchez R.D., Torrescano-Urrutia G.R., González-Ávila M., Rodríguez-Carpena J.G., Huerta-Leidenz N., Pérez-Alvarez J.A., Fernández-López J., Sánchez-Escalante A. (2022). Use of *Pleurotus ostreatus* to Enhance the Oxidative Stability of Pork Patties during Storage and In Vitro Gastrointestinal Digestion. Foods.

[92] Botella-Martínez C., Muñoz-Tebar N., Lucas-González R., Pérez-Álvarez J.A., Fernández-López J., Viuda-Martos M. (2023). Assessment of Chemical, Physico-Chemical and Sensory Properties of Low-Sodium Beef Burgers Formulated with Flours from Different Mushroom Types. Foods.

[93] Tabaldo-Tucar M., Solar M.N.J.B. (2019). Choose Your Patty: The Sensory Characterization and Consumer Acceptance of Burger Patties with Oyster Mushroom (*Pleurotus ostreatus*) Enrichment. J. Phys. Conf. Ser..

[94] Tokarczyk G., Felisiak K., Adamska I., Przybylska S., Hrebień-Filisińska A., Biernacka P., Bienkiewicz G., Tabaszewska M. (2023). Effect of Oyster Mushroom Addition on Improving the Sensory Properties, Nutritional Value and Increasing the Antioxidant Potential of Carp Meat Burgers. Molecules.

[95] Cerón-Guevara M.I., Rangel-Vargas E., Lorenzo J.M., Bermúdez R., Pateiro M., Rodríguez J.A., Sánchez-Ortega I., Santos E.M. (2020). Reduction of Salt and Fat in Frankfurter Sausages by Addition of *Agaricus bisporus* and *Pleurotus ostreatus* Flour. Foods.

[96] Boylu M., Hitka G., Kenesei G. (2024). Sausage Quality during Storage under the Partial Substitution of Meat with Fermented Oyster Mushrooms. Foods.

[97] Cerón-Guevara M.I., Santos E.M., Lorenzo J.M., Pateiro M., Bermúdez-Piedra R., Rodríguez J.A., Castro-Rosas J., Rangel-Vargas E. (2021). Partial Replacement of Fat and Salt in Liver Pâté by Addition of *Agaricus bisporus* and *Pleurotus ostreatus* Flour. Int. J. Food Sci. Technol..

[98] Qing Z., Cheng J., Wang X., Tang D., Liu X., Zhu M. (2021). The Effects of Four Edible Mushrooms (*Volvariella volvacea*, *Hypsizygus marmoreus*, *Pleurotus ostreatus* and *Agaricus bisporus*) on Physicochemical Properties of Beef Paste. LWT.

[99] Abd Rabou E.A.E.-H.A. (2016). The Effect of Substitution Oyster Mushroom on Chemical Composition and Quality Attributes of Meatballs. J. Food Dairy Sci..

[100] If’all I., Nofrianto N., Spetriani S., Sabariyah S., Fathurahmi S., Indriasari Y., Asrawaty A. (2025). Effect of Partial Substitution of Beef with White Oyster Mushroom (*Pleurotus ostreatus*) on Nutritional Profile and Sensory Quality of Meatball Products. J. Health Nutr. Res..

[101] Özünlü O., Ergezer H. (2021). Possibilities of Using Dried Oyster Mushroom (*Pleurotus ostreatus*) in the Production of Beef Salami. J. Food Process. Preserv..

[102] Aritonang S.N., Allismawita A., Dahlia S.N. (2019). The Effect of White Oyster Mushroom (*Pleurotus ostreatus*) Adding on the Quality of Unproductive Quail (*Coturnix japonica*) Abon Shredded Meat. Int. J. Food Sci. Agric..

[103] Ayuso P., García-Pérez P., Nieto G. (2025). New Insights and Strategies in the Nutritional Reformulation of Meat Products Toward Healthier Foods. Molecules.

[104] La A., So A., Ao A., Jo B. (2018). Quality Characteristics of Fortified Bread Produced from Cassava and Mushroom Flours. J. Food Process. Technol..

[105] Irakiza P.N., Chuma G.B., Lyoba T.Z., Mweze M.A., Mondo J.M., Zihalirwa P.K., Mapatano S., Balezi A.Z., Mushagalusa G.N. (2021). Fortification with Mushroom Flour (*Pleurotus ostreatus* (Jacq.) P. Kumm) and Substitution of Wheat Flour by Cassava Flour in Bread-Making: Nutritional and Technical Implications in Eastern DR Congo. Agric. Food Secur..

[106] Oyetayo V.O., Oyedeji R.R. (2017). Proximate and Mineral Composition of Bread Fortified with Mushroom (*Plerotus ostreatus* and *Calocybe indica*). Microbiol. Res. J. Int..

[107] Losoya-Sifuentes C., Simões L.S., Cruz M., Rodriguez-Jasso R.M., Loredo-Treviño A., Teixeira J.A., Nobre C., Belmares R. (2022). Development and Characterization of *Pleurotus ostreatus* Mushroom—Wheat Bread. Starch/Staerke.

[108] Stępniewska S., Salamon A., Cacak-Pietrzak G., Piecyk M., Kowalska H. (2025). The Impact of Oyster Mushrooms (*Pleurotus ostreatus*) on the Baking Quality of Rye Flour and Nutrition Composition and Antioxidant Potential of Rye Bread. Foods.

[109] Ndung’U S.W., Otieno C.A., Onyango C., Musieba F. (2015). Composition of Polyphenols in Wheat Bread Supplemented with *Pleurotus ostreatus* Mushroom. Am. J. Food Technol..

[110] Mishra A. (2022). Modification and Development of Oyster Mushroom-Based Cookies. Emerg. Trends Nutraceuticals.

[111] Carrasco-González J.A., Serna-Saldívar S.O., Gutiérrez-Uribe J.A. (2017). Nutritional Composition and Nutraceutical Properties of the Pleurotus Fruiting Bodies: Potential Use as Food Ingredient. J. Food Compos. Anal..

[112] Devi P.V., Narzary P., Sharma D., Islam J., Sultana F. (2024). Effect of Oyster Mushroom on Nutritional Composition, Shelf Life and Organoleptic Characteristics of Enriched Cookies. Food Humanit..

[113] Sławińska A., Jabłońska-Ryś E., Gustaw W. (2024). Physico-Chemical, Sensory, and Nutritional Properties of Short-bread Cookies Enriched with *Agaricus bisporus* and *Pleurotus ostreatus* Powders. Appl. Sci..

[114] Uriarte-Frías G., Hernández-Ortega M.M., Gutiérrez-Salmeán G., Santiago-Ortiz M.M., Morris-Quevedo H.J., Meneses-Mayo M., Lumumba P. (2021). Pre-Hispanic Foods Oyster Mushroom (*Pleurotus ostreatus*), Nopal (*Opuntia ficus-indica*) and Amaranth (*Amaranthus* sp.) as New Alternative Ingredients for Developing Functional Cookies. J. Fungi.

[115] Wang L., Brennan M.A., Guan W., Liu J., Zhao H., Brennan C.S. (2021). Edible Mushrooms Dietary Fibre and Antioxidants: Effects on Glycaemic Load Manipulation and Their Correlations Pre- and Post-Simulated in Vitro Digestion. Food Chem..

[116] Kondyli E., Pappa E.C., Arapoglou D., Metafa M., Eliopoulos C., Israilides C. (2022). Effect of Fortification with Mushroom Polysaccharide β-Glucan on the Quality of Ovine Soft Spreadable Cheese. Foods.

[117] Kondyli E., Pappa E.C., Kremmyda A., Arapoglou D., Metafa M., Eliopoulos C., Israilides C. (2020). Manufacture of Reduced Fat White-Brined Cheese with the Addition of β-Glucans Biobased Polysaccharides as Textural Proper-ties Improvements. Polymers.

[118] Sołowiej B.G., Nastaj M., Waraczewski R., Szafrańska J.O., Muszyński S., Radzki W., Mleko S. (2023). Effect of Polysaccharide Fraction from Oyster Mushroom (*Pleurotus ostreatus*) on Physicochemical and Antioxidative Properties of Acid Casein Model Processed Cheese. Int. Dairy J..

[119] Rahman A., Roy J., Mahomud S. (2023). Textural and Antioxidant Properties of Mozzarella Cheese Fortified with De-hydrated Oyster Mushroom Flour. Foods Raw Mater..

[120] Radzki W., Skrzypczak K., Sołowiej B., Jabłońska-Ryś E., Gustaw W. (2023). Properties of Yogurts Enriched with Crude Polysaccharides Extracted from *Pleurotus ostreatus* Cultivated Mushroom. Foods.

[121] Sakul S., Komansilan S., Tamasoleng M., Rumondor D., Hadju R. (2024). Antioxidant Activity and Viability Lactoba-cillus Acidophilus Synbiotic Yogurt with the Addition of White Oyster Mushroom (*Pleurotus ostreatus*) Extract. IOP Conf. Ser. Earth Environ. Sci..

[122] Pelaes Vital A.C., Goto P.A., Hanai L.N., Gomes-da-Costa S.M., de Abreu Filho B.A., Nakamura C.V., Matumoto-Pintro P.T. (2015). Microbiological, Functional and Rheological Properties of Low Fat Yogurt Supplemented with *Pleurotus ostreatus* Aqueous Extract. LWT—Food Sci. Technol..

[123] Shams E.A., Yousef N.S., El-Shazly H.A.M., Mark C. (2020). Chemical, Sensory and Rheological Evaluation of Karish Cheese Made by Oyster Mushroom Mycelium. J. Food Dairy Sci. J..

[124] Moshtaghian H., Parchami M., Rousta K., Lennartsson P.R. (2022). Application of Oyster Mushroom Cultivation Residue as an Upcycled Ingredient for Developing Bread. Appl. Sci..

[125] Bermúdez-Gómez P., Muñoz-Tébar N., Martínez-Navarrete G., Lucas-Gonzalez R., Pérez-Clavijo M., Fernández E., Fernández-López J., Viuda-Martos M. (2025). Effect of Sodium and Starch Reduction on Nutritional, Physico-chemical, and Sensorial Properties of Mortadella Added with *Agaricus bisporus* and *Pleurotus ostreatus* by-Products. Meat Sci..

[126] Abu El-Maaty A.S.M., El-Nemr S.E., El-Shourbagy G.A., Galal G.A. (2016). Effect of addition oyster mushroom and red beet root by-products on quality of pan bread. Zagazig J. Agric. Res..

[127] dos Santos J.F., de Oliveira N.M.T., da Silva Milhorini S., Rutckeviski R., Iacomini M., de Souza L.M., Simas F.F., Maria-Ferreira D., Smiderle F.R. (2025). The Use of *Pleurotus ostreatus* By-Products for the Preparation of a Gel-like Polysaccharide with Bioactive Properties. Int. J. Biol. Macromol..

[128] Baptista F., Almeida M., Paié-Ribeiro J., Barros A.N., Rodrigues M. (2023). Unlocking the Potential of Spent Mushroom Substrate (SMS) for Enhanced Agricultural Sustainability: From Environmental Benefits to Poultry Nutrition. Life.

[129] Lekmine S., Boussekine S., Akkal S., Martín-García A.I., Boumegoura A., Kadi K., Djeghim H., Mekersi N., Bendjedid S., Bensouici C. (2021). Investigation of Photoprotective, Anti-Inflammatory, Antioxidant Capacities and LC–ESI–MS Phenolic Profile of *Astragalus gombiformis* Pomel. Foods.

[130] Nieto G., Bañon S., Garrido M.D. (2012). Incorporation of thyme leaves in the diet of pregnant and lactating ewes: Effect on the fatty acid profile of lamb. Small Rumin. Res..

[131] Segueni N., Boutaghane N., Asma S.T., Tas N., Acaroz U., Arslan-Acaroz D., Shah S.R.A., Abdellatieff H.A., Akkal S., Penalver R. (2023). Review on propolis applications in food preservation and active packaging. Plants.

